# Post-exposure serological responses to malaria parasites in potential blood donors

**DOI:** 10.1186/s12936-016-1586-x

**Published:** 2016-11-09

**Authors:** Daniela Portugal-Calisto, Ana Raquel Ferreira, Marcelo Sousa Silva, Rosa Teodósio

**Affiliations:** 1 Global Health and Tropical Medicine, GHTM, Instituto de Higiene e Medicina Tropical, IHMT, Universidade Nova de Lisboa, UNL, Rua da Junqueira 100, 1349-008 Lisbon, Portugal; 2 Instituto Português do Sangue e da Transplantação, Parque de Saúde de Lisboa, Av. do Brasil, 53-Pav. 17, 1749-005 Lisbon, Portugal; 3 Department of Clinical and Toxicological Analysis, Faculty of Pharmacy, Universidade Federal do Rio Grande do Norte, Campus Universitário Lagoa Nova, Natal, 59078-970 Brazil; 4 Programa de Pós-graduação em Bioquímica, Universidade Federal do Rio Grande do Norte, Campus Universitário Lagoa Nova, Natal, 59078-970 Brazil

**Keywords:** Malaria, *Plasmodium* spp., Anti-*Plasmodium* spp. antibodies, Longevity, Blood transfusion

## Abstract

**Background:**

Cases of transfusion-transmitted malaria have been described around the world and highlighted in some studies. Semi-immune individuals are more likely to transmit malaria as they may be asymptomatic. Some countries allow blood donations only based on epidemiological criteria while others reinforce their criteria with serological tests. However, little is known about the longevity of anti-*Plasmodium* spp. antibodies and its meaning in blood donation. Therefore, this study aims to assess the longevity of different subclasses of anti-*Plasmodium* spp. antibodies in individuals with previous stays in endemic areas, as well as to assess how those antibodies are related to personal features and travel characteristics. Based on those results, the suitability of the Portuguese blood donors screening method was addressed, i.e. the method to search for an eventual risk of transfusion–transmitted malaria among the population studied.

**Results:**

Statistical associations were found between the presence of total anti-*Plasmodium* spp. antibodies and some travel characteristics, namely to be born in endemic area versus non endemic and previous episodes of malaria. The intersection between seropositive results and the last year of stay in endemic areas showed a longer longevity of anti-*Plasmodium* spp. antibodies than previously reported. Those results represented a considerable portion of the individuals having returned from their last stay in endemic areas more than 10 years before enrolment in this study. Considering the study population as potential blood donors, serological results also indicated that if epidemiological criteria alone were applied to screen blood donors, an important percentage of seropositive individuals would be approved for blood donation. Because the nature and meaning of those antibodies in the blood donation context is still not understood, those approved individuals could represent a risk for blood transfusion safety.

**Conclusions:**

The place of birth and past episodes of malaria seem to be related to the serological outcome. Epidemiological criteria to screen potential blood donors are insufficient to guarantee the safety of the blood, if applied alone.

**Electronic supplementary material:**

The online version of this article (doi:10.1186/s12936-016-1586-x) contains supplementary material, which is available to authorized users.

## Background

Malaria is an infectious disease with five species of *Plasmodium* infective to humans. Malaria is endemic in tropical and sub-tropical regions, being the sub-Saharan region the most affected, where *Plasmodium falciparum* is the most prevalent species [1].

Since a protective immune response against *Plasmodium* infection is strain-specific, several infections are required in order to contact with a wide repertoire of *Plasmodium* antigens and fulfil the compartment of immunologic memory [2]. A protective immunity against the infection is rare or never achieved. However individuals may become semi-immune to the disease, living in balance with the parasite and carrying it in their bloodstream without symptoms [3].

Some studies have suggested that when individuals leave endemic areas for some period of time, semi-immunity may be lost [4]. However, little is known about antibody dynamics in the context of malaria and the period of time needed for seroreversion is not well determined. A study of Fowkes et al. which was based on a mathematical model, estimated antibodies half-life of 7.6 years in a population of pregnant women [5]. Faddy et al. studied a population of blood donors and concluded that antibodies anti-*Plasmodium* spp. may persist in the bloodstream up to 19.6 months after the last exposure [6]. In addition, the majority of the research work published cannot be analysed in the context of the longevity of anti-*Plasmodium* spp. antibodies: (a) many studies focus on children populations, whose immune system is still in development and thus, it may not be appropriate to extrapolate conclusions to adult populations [7, 8]; (b) some studies were performed in endemic areas where people are constantly at risk of being infected [8, 9] and thus, data about the last exposure and antibody longevity may not be accurate; (c) some studies focus mainly on the existence of parasitaemia at the time of blood collection [10, 11], and (d) some studies only focus on a small number of parasite proteins that generate a humoral response in vaccine studies [12]. Therefore, to better understand the longevity of malaria antibodies or which features may influence it, a study was conducted in a naturally-exposed adult population that is no longer exposed to the parasite. Since semi-immune individuals may live in balance with the parasite without symptoms, these people are more likely to transmit infection through blood transfusion [3, 13].

Transfusion-transmitted malaria has been reported globally, involving children and adults [14–20]. In order to avoid that problem, several governments and other organizations, including the Council of Europe or World Health Organization, created a set of policies and recommendations establishing conditions that shall be applied to potential blood donors [21–23]. In endemic countries, where people are commonly semi-immune, the parasite loads may be undetectable with the available detection techniques. Therefore, transfusion strategies focus on chemoprophylaxis for the donor and recipient, or ensure that the blood collected in areas of high endemicity is not transfused to recipients of low endemicity areas [24]. Despite the efforts, donations frequently end up with the infection of the recipient [20, 25–28]. On the other hand, in nonendemic countries, the strategy to minimize the risk of transmitting malaria through blood transfusion is based on the probability of an individual to transmit the parasite at the moment of blood donation. Therefore, some countries only apply selective epidemiological questionnaires (e.g. Canada, USA) [29–31], while others reinforce their selection measures with immunological tests and/or molecular techniques (e.g. Australia, France, England, Italy, Spain and Portugal [21, 32–36]. The Portuguese Institute for Blood and Transplantation bases its procedures on a Portuguese statement that regulates blood donations for individuals with stays in malaria-endemic areas, who wish to donate blood [21–23, 37]; Table 1 summarizes the Portuguese criteria for donors approval.

**Table 1:** Criteria for donor blood screening, according to the Portuguese Institute for Blood and Transplantation

Risk category	Guidelines
Individuals who lived the first 5 years of life in endemic areas	Approved 3 years after the last stay in endemic areas, since asymptomatic or approved 4 months after return if serological or molecular tests are negative
Previous episodes of malaria	Approved 3 years after cessation of last symptoms/treatment and only if serological or molecular tests are negative
Asymptomatic visitors (stays < 6 months)	Approved 1 year after returning of endemic areas or approved if serological or molecular tests are negative
Febrile episodes undiagnosed during or 6 months after a travel to endemic areas	Approved 3 years after returning from endemic areas and cessation of symptoms or approved 4 months after return if serological or molecular tests are negative

The historical and cultural proximity between Portugal and some endemic countries, such as Angola, Guinea-Bissau, São Tomé and Príncipe, Mozambique, and Brazil, makes Portugal at risk of receiving semi-immune people that later may donate blood [38]. Besides, there are no estimates about the prevalence of individuals with anti-*Plasmodium* spp. antibodies in Portugal. Thus, the present work aims to better understand the dynamics of anti-*Plasmodium* spp. antibodies in a population of adults, naturally-exposed to *Plasmodium* spp. at some moment of their lives. Specifically, this study aims to determine the longevity of anti-*Plasmodium* spp. antibodies; how that longevity may be related to demographic characteristics and travel features; and, according to that, a question about the suitability of the screening method of blood donors was raised, regarding the substantial need of blood units versus the potential risk of transmitting malaria.

## Methods

### Study population

The population studied included 505 individuals that fulfil the following criteria: (1) age between 18 and 65 years old; (2) have stayed in endemic areas of malaria, independently of having a history of malaria episodes, length of stay or reason for travelling.

All individuals that attended different phlebotomy services in Lisbon, between September 2010 and January 2011, and between March and July 2014, and fulfilled the criteria above were invited to participate. Individuals with laboratory diagnostic of malaria at the time of blood collection were excluded and directed to medical experts for follow-up. Any other pathology that people might have had did not interfere with the serologic test used. People included were considered as potential blood donors. An informed consent and a signed permission to give 3 ml of blood for malaria studies were applied to all subjects at the time of enrolment. A questionnaire assessing the risk of exposure was also given to all subjects included. This study was approved by the Ethical Council of the Institute of Hygiene and Tropical Medicine of Lisbon, Portugal.

### Sample collection

Total blood was collected by venepuncture into 3 ml ethylenediamine-tetraacetate (EDTA) tubes. 15 µl of total blood was absorbed in filter paper discs of 11 mm (Whatman, USA), allowed to dry at room temperature for 24 h and stored at 4 °C. The remaining sample was centrifuged at 4000*g* for 15 min, and plasma was stored at −20 °C.

### Serological measurement of anti-*Plasmodium* spp. antibodies

Total anti-*Plasmodium* spp. antibodies were searched in 505 samples, by a commercial immunoenzymatic assay named EIA Malaria Kit Test (Bio-Rad, USA), following the manufacturer’s instructions. This test is used in the Portuguese Institute for Blood and Transplantation to screen potential blood donors regarding the risk of transmitting malaria by blood transfusion.

The immunoassay uses four recombinant proteins immobilized on a solid phase where anti-*Plasmodium* spp. antibodies will bind if present in the serum/plasma sample. Three of the four recombinant proteins are specific to *P. falciparum*, with cross-reactivity for *Plasmodium malariae* and *Plasmodium ovale*, and the fourth recombinant protein is specific for *Plasmodium vivax*. These proteins can detect total immunoglobulins (Ig) from class G, M and A, although not separately.

The threshold was obtained by calculating the quotient of the optical density (OD; measured for each sample) and the cut-off, which is the mean of the negative controls for each plate plus a ponderation of 0.1 (as indicated by the manufacturer). This method allowed reproducible and comparable results between assays. Thus, samples with an OD/cut-off lower than 0.9 were considered negative while samples above 1.1 were considered positive. Samples with values 10% upper or 10% down 1.0 (ranging from 0.91 to 1.09) were repeated and, when the value persisted, were considered inconclusive.

### DNA extraction and amplification

Polimerase chain reaction (PCR) was performed to verify if seropositive results were due to acute infections. Plasmodial genomic DNA was extracted from dried bloodspots of the seropositive samples and negative controls, using Chelex-100 (Bio-Rad, USA), following a protocol adapted from Kain and Lanar’s work [39]. Approximately 1/4 of a 6 mm diameter filter paper with blood absorbed was excised, corresponding to approximately 5 µl of sample. Each excised filter paper was added to a 1.5 ml microcentrifuge tube with 300 µl 10% (w/v) Chelex-100 and vortexed for 15 s, followed by a short spin. Tubes were placed in a heating block at 95 °C for 20 min. Samples were vortex for 15 s and a new short spin was made. The supernatant was transferred into a new 1.5 ml microcentrifuge tube and stored at −20 °C until required for DNA amplification.

PCR reactions were performed for all subjects that were positive in the ELISA technique. DNA of *P. falciparum*, *P. vivax*, *P. malariae* and *P. ovale* was amplified by nested-PCR, using primers that recognize the small subunit (18S) ribosomal RNA gene, designed by Snounou et al. [40]. For amplification, the protocol was adapted from the manufacturer’s instructions of MyTaq Blood-PCR Kit (Bioline, UK). Thus, for each PCR reaction were used 12.5 µl of MyTaq Blood-PCR Mix, 2x; 1 µl of 10 µM forward primer; 1 µl of 10 µM reverse primer; 1 µl of DNA extracted and DEPC-treated water (DNAses and RNAses free) (Bioline, UK) up to 25 µl.

Amplification conditions were: (a) initial denaturation at 94 °C for 2:00 min; (b) denaturation at 94 °C for 0:30 min; (c) annealing at 55 °C for 1:30 min; (d) extension at 72 °C for 2:00 min; repeat step (b) to (d) for 40 cycles; (e) final extension at 72 °C for 10:00 min. DNA extraction was accompanied by a negative control (a piece of blank filter paper) and additionally, every PCR run included a negative control to which no template was added.

### Questionnaire

Subjects included were asked to fill in a questionnaire about personal characteristics such as age, country of birth and years of continuous residence in that country after birth; and travel features namely length of stay in endemic areas of malaria; length of time since the last stay and previous history of malaria. Since epidemiological studies using questionnaires are susceptible to errors due to recalls, the last stay in endemic areas was considered to be the most reliable moment from which there was no more risk of exposure to *Plasmodium* sp. and thus, the last possibility of having a new infection that may justify the antibodies detected at the time of this study. That variable is preferred over the last episode of malaria for several reasons: (1) a person may remain at risk of infection after the disease, every time he/she is in an endemic area; (2) a person may harbour parasites asymptomatically and (3) because the last episode of malaria adds more uncertainty concerning to when it occurred.

The “length of stay” is the duration of the last travel. A short stay was defined as a period of time spent in endemic areas of less than 6 months; and a long stay was considered a period of time of 6 or more months continuously spent in endemic areas. The variable “length of time since the last stay” refers to the time passed since the moment that a person leaves endemic areas until the moment of enrolment in this study. It was categorized according to the criteria for blood donation in Portugal (Table 1).

A pre-test of the questionnaire was made. Since technical questions were not asked, a self-administered questionnaire was applied. The questionnaire included closed, single‐choice questions and open questions.

### Statistical analysis

Quantitative variables were described using mean and standard deviation (SD) or median. Data were analysed using descriptive statistic and hypothesis tests such as Chi Square test of independence, and Mann–Whitney U test for independent samples [41, 42]. A statistical significance level of 5% was used to search for statistical associations or differences between the variables studied. Only the variables statistically significant (p < 0.05) in bivariate analysis were maintained in the second model. Multi-variate analysis was performed in two steps: a first one using a univariate logistic regression and a second step using multiple logistic regression, based on forward likelihood ratio method. The quality of the adjustment of the model was assessed using Hosmer and Lemeshow Test. The hypothesis H_0_ was excluded when p < α (α = 0.05). The adjustment of the logistic regression model was also performed using a statistical significance level of 5%. Statistical analyses were performed with IBM SPSS Statistics version 20.0 software (IBM SPSS Statistics for Windows, Version 20.0. Armonk, NY: IBM Corp.).

## Results

### Characteristics of the studied population

This study included 505 individuals with previous stays in endemic areas of malaria, of which 336/505 were men (66.5%) and 169/505 were women (33.5%). The mean age was 41.34 years (95% confidence interval: 40.27–42.41), SD = 12.229, ranging from 18 to 65 years old. The median age was 40 years old. The most represented nationalities were Portugal (73.3%), followed by Angola (13.1%) and Mozambique (5.8%). Overall, approximately 75.8% of the individuals were born in a nonendemic malaria country. Table 2 summarizes the frequency of the variables studied.

**Table 2:** Frequency of travel characteristics and malaria history and their statistical relations with total anti-*Plasmodium* spp. antibodies

	Presence of antibodies					Crude logistic regression^a^				Adjusted logistic regression^a^			
	Overall n (%)	Yes n (%)	No n (%)	χ^2^	p value	β_*i*_	p value	OR	CI 95%	β_*i*_	p value	OR	CI 95%
Birth in endemic areas n = 495													
Yes	120 (24.2)	35 (53.0)	85 (19.8)	34.36	<0.001	1.519	<0.001	4.569	(2.667;7.830)	0.843	0.011	2.324	(1.209;4.464)
No	375 (75.8)	31 (47.0)	344 (80.2)			**–**	**–**	Reference category	–	–	–	Reference category	–
First 5 years of life in endemic area n = 86													
Yes	75 (87.2)	24 (92.3)	51 (85.0)	0.89	0.351	0.739	0.365	2.095	(0.423;10.363)	^b^			
No	11 (12.8)	2 (7.7)	9 (15.0)			**–**	**–**	Reference category	–				
Previous history of malaria n = 485													
Yes	165 (34.0)	51 (79.7)	114 (27.1)	68.50	<0.001	2.358	<0.001	10.565	(5.539;20.152)	2.183	<0.001	8.872	(4.344;18.118)
No	320 (66.0)	13 (20.3)	307 (72.9)					Reference category	–	–	–	Reference category	–
							0.788^d^						
Number of travels to endemic areas n = 480													
1	220 (45.8)	25 (44.6)	195 (40.6)	0.48	0.787	−0.246	0.541	0.782	(0.356;1.719)	^b^			
2–9	189 (39.4)	21 (37.5)	168 (39.6)			−0.271	0.511	0.763	(0.340;1.711)				
≥10	71 (14.8)	10 (17.9)	61 (14.4)			**–**	**–**	Reference category	–				
Length of stay n = 476													
<6 month	306 (64.3)	20 (37.0)	286 (67.8)	19.69	<0.001	−1.274	<0.001	0.280	(0.155; 0.504)	^c^			
≥6 month	170 (35.7)	34 (63.0)	136 (32.2)			**–**	**–**	Reference category	–				
Length of time since last stay n = 502							0.274^d^						
<4 month	213 (42.4)	34 (50.7)	179 (41.1)	4.06	0.255	0.204	0.478	1227	(0.697;5.128)	^b^			
4 month–1 year	50 (10.0)	5 (7.5)	45 (10.3)			−0.332	0.523	0.718	(0.259;1.988)				
1–3 years	60 (12.0)	4 (6.0)	56 (12.9)			−0.774	0.169	0.461	(0.153;1.388)				
≥3 years	179 (35.7)	24 (35.8)	155 (35.6)			**–**	**–**	Reference category	–				
Age	–	–	–	–	–	0.037	0.001	1.038	(1.016;1.060)	^c^			

β_*i*_ coefficient of the independent variables in the model

*OR* odds ratio; CI 95–95% of confidence interval for odds ratio

p value statistical significant <0.05

^a^Dependent variable: presence of antibodies

^b^Variable not included in the model, because in bivariate analysis it was not statistically significant

^c^Variable excluded from the model, by method forward likelihood ratio (LR)

^d^p value common to all the categories within the same variable

Although 120 respondents were born in endemic areas of malaria, only 75 individuals lived the first 5 years of life in the endemic areas where they were born. Regarding previous episodes of the disease, “no previous history of malaria” was the answer for the majority of the respondents (Table 2). From the individuals that answered “having history of malaria” (165/505), only 38.6% (63/163) of individuals were born in endemic areas of malaria against to 61.3% (100/163) not born in endemic areas (Additional file 1).

Regarding the amount of travels to endemic areas of malaria, there are more people that travelled only once, contrarily to people who travelled more than 10 times to endemic areas, who accounts for the minor subpopulation. For the majority of the respondents, the length of stay in endemic areas was less than 6 months. Relatively to the length of time since the last stay in endemic areas, there are two subpopulations that stands out: one returning 4 months before their enrolment and another subpopulation that returned more than three years before the study (Table 2), of which 63% (113/179) had their last stay to endemic areas more than 10 years before their enrolment (Additional file 2).

### Serological profile and DNA detection

**Fig. 1:**
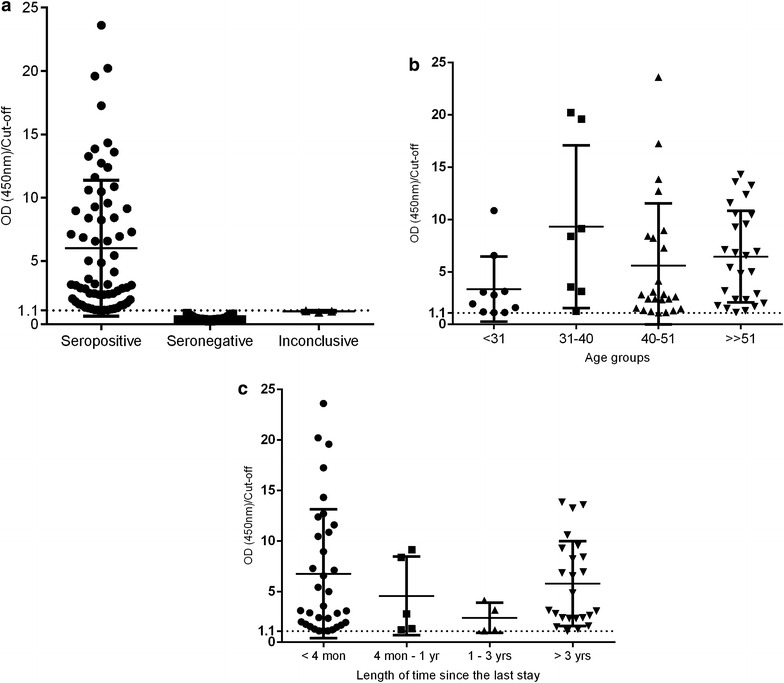
Distribution of levels of total anti-*Plasmodium* spp. antibodies, according to **a** ELISA results: seropositive (n = 67); seronegative (n = 435) and inconclusive (n = 3); **b** distribution of levels of total anti-*Plasmodium* spp. antibodies of the seropositive group, according to age groups: <31 years old (10/67; 14.9%); 31–40 years old (7/67; 10.4%); 40–51 years old (24/67; 35.8%); ≥51 years old (26/67; 38.8%); **c** Distribution of levels of total anti-*Plasmodium* spp. antibodies of the seropositive group, according to the length of time since the last stay in endemic areas of malaria: <4 months (34/67; 50.7%); 4 months–1 year (5/67; 7.5%); 1–3 years (4/67; 6%); ≥3 years (24/67; 35.8%)

The prevalence and level of total anti-*Plasmodium* spp. antibodies were determined with EIA Malaria Kit Test (Bio-Rad, USA). According to the serological results, 67 individuals (13.3% of the total) were seropositive against *Plasmodium* spp. and 435 individuals (86.1%) were seronegative against the parasite [three results (0.6%) were inconclusive]. The distribution of the levels of total anti-*Plasmodium* spp. antibodies among the population studied (n = 505) are presented in Fig. 1a.

**Fig. 2:**
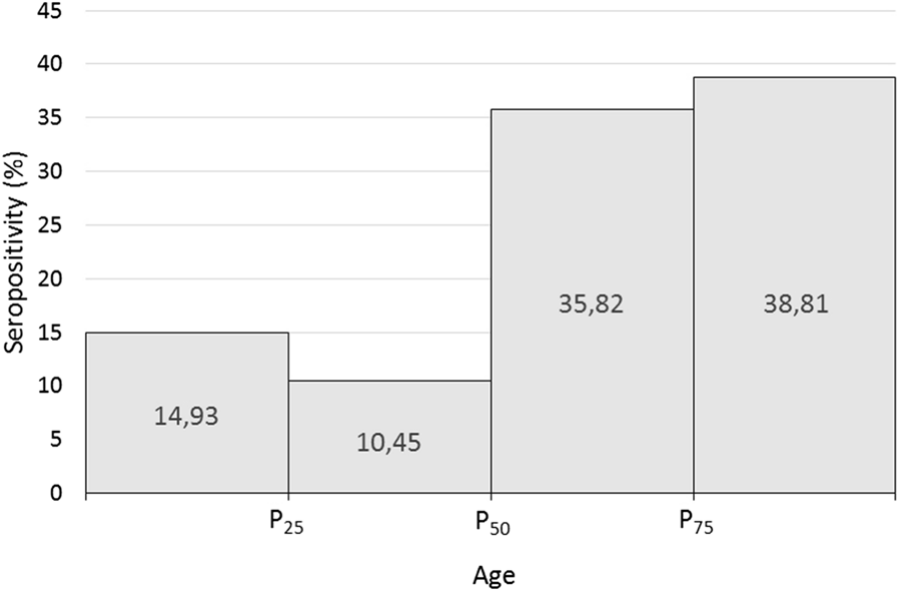
Percentage of seropositive individuals according to the presence of total antibodies anti-*Plasmodium* spp. in each percentile. Variable “Age” categorized according to P_25_, P_50_ and P_75_, resulting in four age groups: <31 years old (10/67; 14.9%); 31–40 years old (7/67; 10.4%); 40–51 years old (24/67; 35.8%); ≥51 years old (26/67; 38.8%)

In order to understand how total anti-*Plasmodium* spp. antibodies are present in the population studied, we analysed the distribution of the levels of those antibodies according to age group and the length of time since the last stay in endemic areas of malaria (Fig. 1b, c, respectively). The variable “age” was categorized according to percentiles P_25_ = 31 years old; P_50_ = 40 years old and P_75_ = 51 years old. The percentage of seropositivity per percentile is presented in Fig. 2. The groups with more percentage of seropositive individuals correspond to the percentiles of P_50_ and P_75_, being also the subgroups with a higher value of OD/cut-off. Regarding the length of time since the last stay in endemic area, there are more seropositive individuals returned 4 months before their participation in the study and returned 3 or more years before their participation (Fig. 1c).

In order to distinguish between acute infections from old infections in seropositive subjects, and to help to assure that the seropositivity detected is not due to circulating parasites, PCR reactions were made. Samples with *Plasmodium* DNA were excluded and thus, all subjects included were negative in PCR at the time of blood collection.

### Statistical relation between the variables studied and total anti-*Plasmodium* spp. antibodies

Individual and travel features that could justify the longevity of total anti-*Plasmodium* spp. antibodies were searched and statistically analysed. In the bivariate statistical analysis, the presence of total antibodies anti-*Plasmodium* spp. is statistically associated with the length of stay in endemic areas (N = 476, χ^2^ = 19.69, p < 0.001) (Table 2). Notwithstanding, the level of total antibodies seems to be higher in individuals that stayed in endemic areas for periods of time of 6 or more months (Mann–Whitney *U* = 29.90, p < 0.05) (Additional file 3).

Having been born in endemic areas may be an important feature to influence the presence of anti-*Plasmodium* spp. antibodies (N = 495, χ^2^ = 34.36, p < 0.001), such as a previous history of malaria (N = 485, χ^2^ = 68.50, p < 0.001) (Table 2).

The logistic regression model excluded some of the statistical significant variables calculated in the bivariate analysis, indicating that only the variables “Birth in endemic areas” and “Previous history of malaria” were statistically significant, with p = 0.011 and p < 0.001, respectively (Table 2). The odds ratio of having been “Born in endemic areas” was 2.324 (CI_95%_ 1209; 4464) comparatively to ‘Not born in endemic areas’, which means that having been born in an endemic area increases in 132.4% (2.324 − 1×100) the possibility of having antibodies. Notwithstanding, the odds ratio of having a previous history of malaria was 8.872 (CI_95%_ 4344; 18,118) comparatively to not have a previous history of malaria. In this case, a previous history of malaria increases in 878.2% the probability of an individual be seropositive (Table 2). The Hosmer and Lemeshow test, with a *p* value of 0.027, led to the rejection of the null hypothesis and to the conclusion that the model does not fit well the data (Additional file 4).

**Fig. 3:**
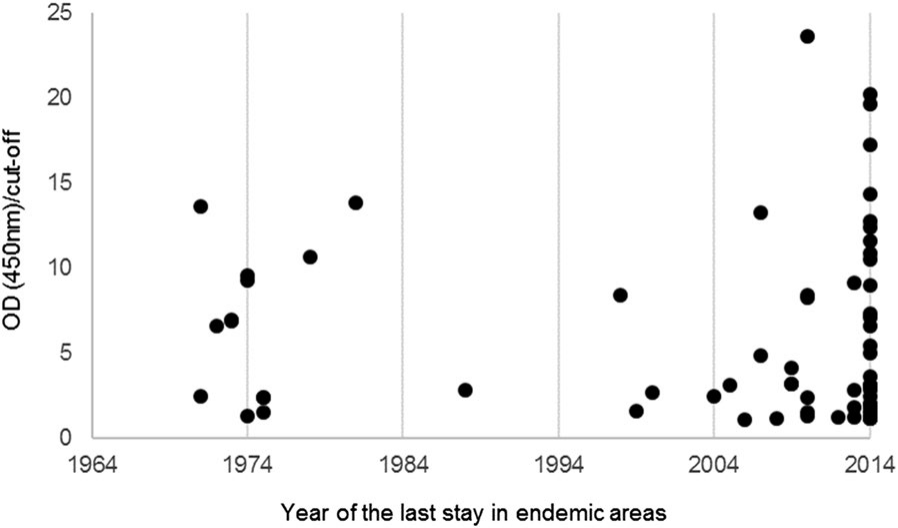
Distribution of antibody levels among the seropositive group, according to the year of their last stay in endemic areas of malaria

Regarding to the last stay in endemic areas of malaria, and comparing the year of the last exposure with the level of anti-*Plasmodium* spp. antibodies, two seropositive groups stand out: individuals returned in the last 2 years and another important population returned from endemic areas more than 10 years before their enrolment. The oldest exposures among seropositive individuals occurred 43 years before their participation in the study (Fig. 3).

### Implication of *anti*-*Plasmodium spp.* antibodies in blood donation

Considering that all individuals were negative in PCR and thus, considered as potential blood donors, the percentage of donors approved for blood donation was assessed, according to the Portuguese criteria for blood donors screening. Table 4 shows that if epidemiological criteria were applied alone to screen blood donors, there would be a risk of seropositive individuals be approved for blood donation. On the other hand, when laboratory criteria were applied in combination with the epidemiological criteria, the percentage of individuals approved was lower but it did not carry risk for blood donation.

## Discussion

Malaria is a serious condition that still affects many people worldwide, being a major cause of transfusion-transmitted diseases [43], and Portugal remains an important destination for people travelling from endemic areas [38, 44, 45]. Asymptomatic people represent an important risk to malaria transmitted through blood transfusion [46, 47]. The existence of anti-*Plasmodium* spp. antibodies in a blood unit may represent a risk of malaria transmission, since it is not clear what their presence means in the context of blood transfusion. As a result, all seropositive individuals for malaria are excluded from blood donations.

The longevity of those antibodies is also not well determined, with several studies indicating different half-life of antibodies anti-*Plasmodium* spp. [5, 6]. Therefore, the effect of malaria exposures in the blood donation context was analysed, treating the subjects included as potential blood donors and thus, applying the Portuguese criteria for donor eligibility. Characteristics that could justify the longevity of the antibodies detected were also searched.

A group of 505 people, ranging from 18 to 65 years old, with previous stays in endemic areas of malaria were included. The majority of the individuals were born in Portugal (a nonendemic area), and thus its immune system was not stimulated against *Plasmodium* antigens during childhood. Negative results from PCR indicate that none of the serological reactivity detected were due to acute infections at the time of enrolment. Overall, 13.3% of the individuals were seropositive for antibodies anti-*Plasmodium* spp. Statistical analysis were performed to search for associations between the presence of total anti-*Plasmodium* spp. antibodies and some personal and travels characteristics (age; country of birth; number of travels to endemic areas; the length of stay in endemic areas and the length of time since the last stay). Results are summarized in Tables 2 and 3.

**Table 3:** Statistical relation between variable “age” and serological results

Variables		Median age (years)	Mean age (years)	Mann–Whitney *U* Test	Mean rank	p value
Age (n = 502)	Group of seropositive (n = 67)	46	46.13 ± 1.46	10.69	309.40	<0.001
	Group of seronegative (n = 435)	39	40.59 ± 0.58		242.58	

The effect of age is not completely understood. Although some studies suggest that a protective immune response is acquired and developed early in life, others indicate that a more mature immune system develops a specific immune response more efficiently [48–50]. The results obtained in the present study suggest that the presence of total anti-*Plasmodium* spp. antibodies is not related to the age of subjects, being a confounding variable of the studied population. However, analysing the percentiles of the variable “age” it is possible to notice that there are more seropositive subjects in the two older percentiles (Fig. 2). The only study founded enrolling an adult population showed a high prevalence of antibodies against *P. vivax* between the ages of 25–50 years [51]. The results reported in the present study indicate that there are more percentage of individuals with total antibodies against *Plasmodium* spp. from 40 to 65 years old than among younger individuals, aged from 18 to 40 years (Fig. 2).

To be born in endemic areas seems to be an important feature that may justify the presence of *anti*-*Plasmodium spp.* antibodies (OR = 2.324 [CI_95%_ 1209; 4464]). Although it has been described that children under 5 years old are one of the most vulnerable groups to malaria infections [52], the present report suggests that living the first 5 years of life in those areas does not seem to be related to the persistence of total anti-*Plasmodium* spp. antibodies until adulthood. Mackinstosh and colleagues analysed the blood of 272 children under 10 years old and indicated the presence of anti-*Plasmodium falciparum* antibodies only in parasitized children, suggesting that those antibodies may be short-lived [53]. To be true that age per se is not a factor that stimulates antibodies that can persist for long periods of time, these results may raise a question about the applicability of some criteria in blood donations.

According to the statistical results obtained in this study, the presence of total antibodies is related to a previous history of malaria (N = 485, χ^2^ = 68.50, p < 0.001), (OR = 8.872 [CI_95%_ 4344; 18,118]), although it does not take into account the moment when it occurred. Those results are consonant with a study of Nguyen including blood donors in the USA, in which the authors analysed the risk of transfusion-transmitted malaria between a group of deferred donors and a group of accepted blood donors [54]. That study suggests that there is a significant risk of transmitting malaria through blood transfusion when people with past episodes of malaria are accepted for donation (without serologic testing) [54]. Although the presence of total anti-*Plasmodium* spp. antibodies seemed to be also dependent of the length of stay in endemic areas (N = 476, X^2^ = 19.69, p < 0.001) according to bivariate analysis, the logistic regression model indicated that this is another confounding variable. Regarding other variables studied, as the number of travels to endemic areas and the time passed since the last stay in endemic areas (as well as the first 5 years of life in endemic areas, already mentioned above), statistical analysis indicate that serological results are independent of those variables, showing no differences statistically significant.

The logistic regression model excluded some of the variables that were statistical significant on the bivariate analysis, indicating that only the variables “birth in endemic areas” and “previous history of malaria” are statistically significant. Therefore, the variables “length of stay” and “age” are possible confounding variables. Although model’s coefficients were statistically significant, Hosmer and Lemeshow test indicated that there was no good quality of model’s adjustment, which can be explained by the existence of other features not included in this study that could better explain the presence and longevity of total anti-*Plasmodium* spp. antibodies.

Although statistical analysis does not give a significant meaning when serological results are analysed regarding the length of time since the last stay (Table 2), the analyses of Fig. 3 shows two important populations that shall be highlighted: one corresponds to individuals who returned from endemic areas within 2 years before the study, and another population showing serological reactivity 40–50 years after the last stay in endemic areas. The two oldest exposures correspond to two people with stays in African countries: one Portuguese man that travelled to Guinea-Bissau for military duties, reported previous episodes of malaria and returned in 1971, with no more stays in endemic areas since. The second person, also a man, was born in Mozambique and lived there for 4 years, between 1967 and 1971, returning 43 years before the study execution, not having history of malaria episodes. The literature is ambiguous about the longevity of anti-*Plasmodium* spp. antibodies, but it can be found that the humoral immune response against *Plasmodium* spp. may be transient, remaining up to 3–5 years without re-exposures [55]. A study of Fowkes et al. including a population of pregnant women in northwestern Thailand, estimated a half-life of 7.6 years for antibodies against PfAMA1 of *P. falciparum* in infected women, although for uninfected participants the longest persistence time calculated was only 3.1 years for MSP2 of *P. falciparum* [5]. Faddy et al. investigated the longevity of antibodies in a population of blood donors in a nonendemic area and indicated a median seroreversion time of 19.6 months. Also, the authors estimated that 20% of people with stays in endemic area of less than 6 months would serorevert 3 years after the initial reactive result, and 2% of the people with stays in endemic areas of more than six months would test seronegative in 5 years [6]. A study of Druilhe et al. reported the presence of antibodies against *P. falciparum* sporozoites in African adults up to 11 years after the last exposure [56]. Therefore, the persistence of antibodies detected in the present report is significantly longer than previously described [5, 6, 56].

The implication of the antibodies detected for blood donation was determined only for two of the four Portuguese blood donation criteria due to its inapplicability: the condition “Individuals with previous episodes of malaria” uses epidemiological and laboratory criteria that are dependent, not allowing a person to donate blood if one of the criteria is not verified, and thus our analyse could not be done. The condition “Individuals with febrile episodes undiagnosed during or 6 months after a travel for endemic areas” cannot be analysed in this study, since the database did not include symptomatic individuals.

As shown in Table 4, epidemiological criteria (which only rely in the length of time since the last stay in endemic areas) approve a higher percentage of potential blood donors than laboratory criteria. However, it carries a risk for blood safety since epidemiological criteria alone would allow the donation of blood from individuals that are seropositive for malaria. Moreover, laboratory criteria are more selective and the percentage of approval is significantly lower. It seems that epidemiological criteria used alone are insufficient to guarantee the blood safety and that laboratory criteria are more reliable once all the individuals approved would be seronegative, and thus the uncertainty about blood safety decreases.

**Table 4:** Percentage of donors approved according to the Portuguese criteria for blood screening and serologic results

Condition		Criteria for approval	Donors approval %	
Individuals who lived the first 5 years of life in endemic areas (n = 85)	Epidemiological criterion	3 year after return (n = 45)	52.9 (45/85)	Of which 28.9% (13/45) that would be approved were seropositive
	Laboratory criterion	If returned between 4 month and 3 year and seronegative (n = 14)	16.5 (14/85)	(All approved would be seronegative)
Asymptomatic travellers (length of stay <6 months) from endemic areas (n = 308)	Epidemiological criterion	1 year after return (n = 147)	47.7 (147/308)	Of which 5.4% (8/147) that would be approved were seropositive
	Laboratory criterion	If returned between 4 month and 1 year with serological test nonreactive (n = 31)	10.1 (31/308)	(All approved would be seronegative)

Despite all efforts made to obtain accurate answers from epidemiological questionnaires, these kind of studies are susceptible to recall bias, which is important to consider when analysing the results.

Serological assays used as screening methods do not necessarily indicate that a person is infected at the moment of the donation, but it constitutes an important indicator to measure the risk of transmitting this infectious disease. In the USA, where the screening of blood donors relies only in epidemiological questionnaires and periods of suspension, it was described a case of transfusion-transmitted malaria caused by *P. malariae* 44 years after exposure in an endemic area of malaria [57]. The present study intends to alert for similar cases, since it was detected anti-*Plasmodium* spp. antibodies many years after exposure and the risk for blood transfusion is not known yet. Thus, measures of eligibility of blood donors should be reinforced. Complementary measures to ensure the safety of the blood may include policies that demand blood screening in areas where those policies do not exist yet, and that blood screening at a laboratory level becomes a regular procedure in every blood donation. Other studies have suggested that the screening of blood donors should include the antigen detection with monoclonal antibodies or the detection of its nucleic acids, in order to reinforce the performance of serologic tests and improve the screening methods and, consequently, improve blood safety [58, 59].

New studies should be performed to deeply investigate molecular characteristics and immunological effects of those antibodies detected decades after exposure. Other subjects or travel characteristics should be considered to contribute to the longevity of anti-*Plasmodium* spp. antibodies. The implication of those antibodies on the criteria of donors eligibility (including the two other requirements for blood donation not contemplated in the present work) should also be assessed in broader and representative populations.

## Conclusions

In the present study, an important percentage of individuals returned from endemic areas more than 10 years before their enrolment was detected with total anti-*Plasmodium* spp. antibodies circulating in their bloodstream.

Some factors that may influence the longevity of total anti-*Plasmodium* spp. antibodies over time were identified: (a) had been born in endemic areas and (b) previous history of malaria. On the other hand, living in endemic areas during childhood does not seem to be related to the longevity of total anti-*Plasmodium* spp. antibodies, as well as the number of travels to endemic areas or the length of time spent in endemic areas, for the population studied. Although the length of time since the last stay in endemic areas was not statistically significant, the presence of total anti-*Plasmodium* spp. antibodies in the bloodstream of individuals many years after exposure, with no history of malaria in the meantime, is important to highlight.

In the blood donation context, epidemiological questionnaires should be reinforced with serological tests to guarantee the safety and quality of the blood donated. New studies should be performed to understand the biological nature of those long-lived immunoglobulins, their specific role in the immunity of malaria and their implication in blood transfusion.

## Additional files

**Additional file 1.** Frequency of individuals born in endemic versus nonendemic area of malaria regarding previous history of malaria.

**Additional file 2.** Frequency of people returned from endemic areas 10 or more years before their enrolment in the study, among the subgroup of subjects returned 3 and more years before enrolment.

**Additional file 3.** Frequency and levels of total anti-*Plasmodium* spp. antibodies comparing the two subgroups of subjects: with a length of stay of less than 6 months and a length of stay of 6 or more months in endemic areas of malaria. The presentation was adapted from the result obtained in Mann-Whitney U test.

**Additional file 4.** Contingency table for Hosmer and Lemeshow Test.
