# Supplementary material for: Post-exposure serological responses to malaria parasites in potential blood donors
Source: Malar J. 2016 Nov 9;15:548. doi: 10.1186/s12936-016-1586-x (PMC5103439; doi:10.1186/s12936-016-1586-x)
Supplement: Supplementary file 1 — Additional file 1. Frequency of individuals born in endemic versus nonendemic area of malaria regarding previous history of malaria. [file 12936_2016_1586_MOESM1_ESM.docx]

Additional file 1. Frequency of individuals born in endemic *versus* nonendemic area of malaria regarding previous history of malaria.

|  | | **Area of Birth** | |
| --- | --- | --- | --- |
|  |  | Non endemic area  n (%) | Endemic area  n (%) |
| **History of Malaria** | Yes (n = 163) | 100 (61.3) | 63 (38.7) |
|  | No (n = 315) | 271 (86.0) | 44 (14.0) |
