# Supplementary material for: Post-exposure serological responses to malaria parasites in potential blood donors
Source: Malar J. 2016 Nov 9;15:548. doi: 10.1186/s12936-016-1586-x (PMC5103439; doi:10.1186/s12936-016-1586-x)
Supplement: Supplementary file 2 — Additional file 2. Frequency of people returned from endemic areas 10 or more years before their enrolment in the study, among the subgroup of subjects returned 3 and more years before enrolment. [file 12936_2016_1586_MOESM2_ESM.docx]

Additional file 2. Frequency of people returned from endemic areas 10 or more years before their enrolment in the study, among the subgroup of subjects returned 3 and more years before enrolment.

|  | **Return from endemic areas ≥ 10 years?** | |
| --- | --- | --- |
|  | Yes  n (%) | No  n (%) |
| Group of people returned from endemic areas 3 or more years before enrolment  (n = 179) | 113 (63.1) | 66 (36.9) |
