# Supplementary material for: Post-exposure serological responses to malaria parasites in potential blood donors
Source: Malar J. 2016 Nov 9;15:548. doi: 10.1186/s12936-016-1586-x (PMC5103439; doi:10.1186/s12936-016-1586-x)
Supplement: Supplementary file 3 — Additional file 3. Frequency and levels of total anti-Plasmodium spp. antibodies comparing the two subgroups of subjects: with a length of stay of less than 6 months and a length of stay of 6 or more months in endemic areas of malaria. The presentation was adapted from the result obtained in Mann-Whitney U test. [file 12936_2016_1586_MOESM3_ESM.docx]

Additional file 3. Frequency and levels of total anti-*Plasmodium* spp. antibodies comparing the two subgroups of subjects: with a length of stay of less than 6 months and a length of stay of 6 or more months in endemic areas of malaria. The presentation was adapted from the result obtained in Mann-Whitney U test.


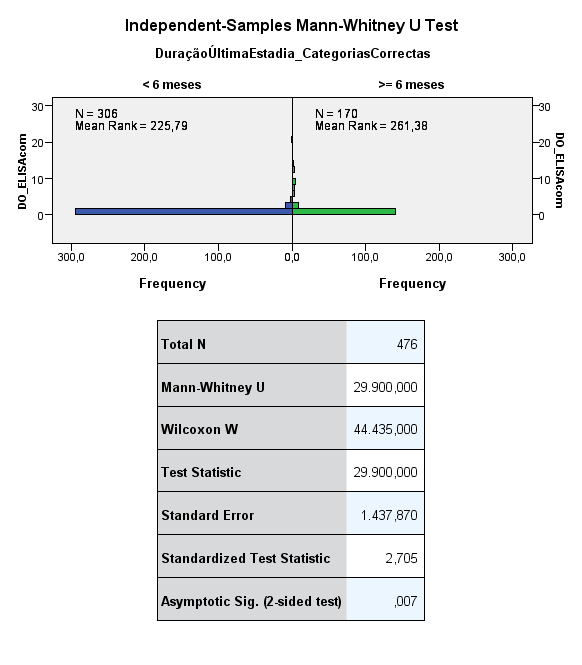


**< 6 months**

OD/cutoff

OD/cutoff

**≥ 6 months**
