# Supplementary material for: Post-exposure serological responses to malaria parasites in potential blood donors
Source: Malar J. 2016 Nov 9;15:548. doi: 10.1186/s12936-016-1586-x (PMC5103439; doi:10.1186/s12936-016-1586-x)
Supplement: Supplementary file 4 — Additional file 4. Contingency table for Hosmer and Lemeshow Test. [file 12936_2016_1586_MOESM4_ESM.docx]

Additional file 4. Contingency table for Hosmer and Lemeshow Test.

| **Groups in final model** | **Presence of antibodies = Negative** | | **Presence of antibodies = Positive** | | **Total** | **Hosmer and Lemeshow Test** | | |
| --- | --- | --- | --- | --- | --- | --- | --- | --- |
|  | Observed | Expected | Observed | Expected |  | Chi-square | df | p-value |
| 1 | 262 | 258,704 | 5 | 8,296 | 267 | 7,199 | 2 | 0,027 |
| 2 | 33 | 36,296 | 6 | 2,704 | 39 |  |  |  |
| 3 | 73 | 76,296 | 25 | 21,704 | 98 |  |  |  |
| 4 | 34 | 30,704 | 17 | 20,296 | 51 |  |  |  |

df- degrees of freedom
